# Effect of Exposed Surface Area, Volume and Environmental pH on the Calcium Ion Release of Three Commercially Available Tricalcium Silicate Based Dental Cements

**DOI:** 10.3390/ma11010123

**Published:** 2018-01-13

**Authors:** Sivaprakash Rajasekharan, Chris Vercruysse, Luc Martens, Ronald Verbeeck

**Affiliations:** 1 Department of Paediatric Dentistry & Special Care, PAECOMEDIS Research Cluster, Ghent University, 9000 Ghent, Belgium; luc.martens@ugent.be; 2 Biomaterials Group, Department of Basic Medical Sciences, Ghent University, 9000 Ghent, Belgium; chris.vercruysse@ugent.be (C.V.); Ronald.Verbeeck@UGent.be (R.V.)

**Keywords:** tricalcium silicate cement, Biodentine, MTA, pH, calcium ion release

## Abstract

Tricalcium silicate cements (TSC) are used in dental traumatology and endodontics for their bioactivity which is mostly attributed to formation of calcium hydroxide during TSC hydration and its subsequent release of calcium and hydroxide ions. The aim of this study was to determine the effect of volume (Vol), exposed surface area (ESA) and pH of surrounding medium on calcium ion release. Three commercially available hydraulic alkaline dental cements were mixed and condensed into cylindrical tubes of varying length and diameter (*n* = 6/group). For the effect of ESA and Vol, tubes were immersed in 10 mL of deionized water. To analyze the effect of environmental pH, the tubes were randomly immersed in 10 mL of buffer solutions with varying pH (10.4, 7.4 or 4.4). The solutions were collected and renewed at various time intervals. pH and/or calcium ion release was measured using a pH glass electrode and atomic absorption spectrophotometer respectively. The change of pH, short-term calcium ion release and rate at which calcium ion release reaches maximum were dependent on ESA (*p* < 0.05) while maximum calcium ion release was dependent on Vol of TSC (*p* < 0.05). Maximum calcium ion release was significantly higher in acidic solution followed by neutral and alkaline solution (*p* < 0.05).

## 1. Introduction

Tricalcium silicate cements (TSC) are generally rich in calcium compounds and form calcium hydroxide during and after the clinical setting period [1,2]. The clinical importance of the formation of calcium hydroxide during the setting of endodontic materials has been emphasized repeatedly in the literature [3]. TSCs are widely used in endodontics and in dental traumatology for pulp capping, pulpotomy, root-end filling, perforation repair, root resorption, apexification and obturation of root canals [4]. Apart from these generally accepted indications, exploratory research is being carried out for the use of newly developed commercial tricalcium silicate based endodontic biomaterials in regenerative endodontic therapy [5], strengthening of weakened endodontically restored teeth in combination with post [6], as a bone substitution material for implant stabilization [7], dentine replacement [8] and temporary restoration of deep caries [9].

These indications are mostly based on the ability of TSCs to form calcium hydroxide upon hydration [10], which subsequently dissociates into calcium and hydroxide ions [11]. Calcium ions activate a series of signaling pathways associated with mineralization [12,13] and hydroxide ions create an alkaline environment responsible for antibacterial and anti-inflammatory activity [14,15]. Quantification of the formation of calcium hydroxide and subsequent release of calcium and hydroxide ions after the setting of these TSCs would provide a better insight into the clinical significance of this release [3].

Numerous tricalcium silicate based cements with subtle variations in composition and manufacturing process are commercially available for endodontic applications. ProRoot^®^ white MTA (Dentsply, Tulsa dental specialties, Tulsa, OK, USA), Medcem MTA^®^ GmbH (Weinfelden, Switzerland) and Biodentine™ (Septodont, Saint Maur des Fosses, France) are endodontic cements with tricalcium silicate as primary ingredient. Mineral Trioxide Aggregate (MTA) as a cement and its clinical applications in endodontics and dental traumatology have been studied extensively with positive results. On the other hand, literature also mentions several drawbacks such as difficult handling, long setting time, possible discoloration, lower compressive and flexural strength compared to dentine [16]. The more recently developed Medcem MTA^®^ is a so-called second generation MTA consisting of purified Portland cement and zirconium oxide instead of bismuth oxide as radiopacifier as the latter is considered responsible for discoloration. Biodentine™ also contains zirconium oxide as radiopacifier and is well-known for its faster setting and superior physical properties [17]. Although tricalcium silicate is the main ingredient, the behavior of these cements can differ due to different radiopacifiers [18], variations in the manufacturing process, purity of the constituents and hydration products [19].

Each clinical application requires an adequate volume of TSC to be used at the site of repair, depending on the severity of the infection. In addition, the surface area of biomaterial exposed to the surrounding oral tissues varies widely. Despite the numerous studies analyzing the pH and calcium ion release of ProRoot^®^ MTA [4,20,21,22,23,24,25,26,27], there has been no research on the effect of environmental pH, volume (Vol) and exposed surface area (ESA) on the release of calcium and hydroxide ions. Moreover, studies of the pH and calcium ion release of Medcem MTA^®^ are lacking. Hence, the aim of the present study was to determine the effect of volume, exposed surface area and the pH of the surrounding moisture on the calcium ion release of three TSCs viz. ProRoot^®^ white MTA, Medcem MTA^®^ and Biodentine™.

## 2. Results

**Figure 1:**
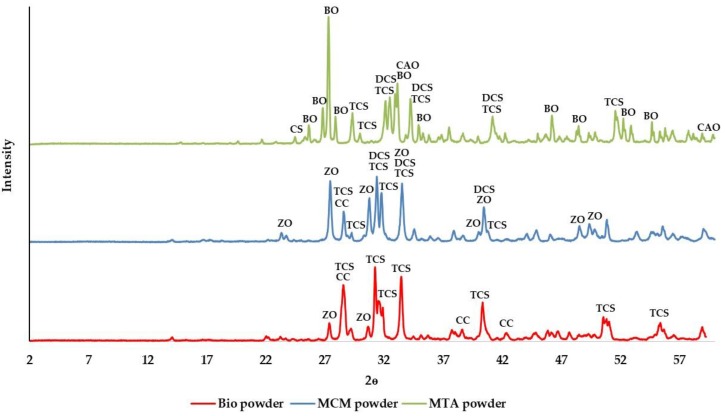
The X-ray diffraction patterns of the powder component of the TSCs studied. Legend: CH: Calcium hydroxide; TCS: Tricalcium silicate; DCS: Dicalcium silicate; CS: Calcium sulphate; ZO: Zirconium oxide; BO: Bismuth oxide; CAO: Calcium aluminium oxide; CC: Calcium carbonate.

X-ray diffraction (XRD) analysis of the powder showed that all the three cements are crystalline (Figure 1). Tricalcium silicate was a common constituent found in ProRoot^®^ white MTA, Medcem MTA^®^ and Biodentine™ but two different forms of tricalcium silicate were identified in these cements. While one crystalline form of tricalcium silicate (Powder Diffraction File 42-551) was found in ProRoot^®^ white MTA and Medcem MTA^®^, Biodentine™ contained a different form of tricalcium silicate (Powder Diffraction File 31-301). The tricalcium silicate present in ProRoot^®^ white MTA and Medcem MTA^®^ was the one with chemical composition closest to that of alite from Portland cement whereas the tricalcium silicate found in Biodentine™ was synthesized. In addition to tricalcium silicate, dicalcium silicate was identified only in ProRoot^®^ white MTA and Medcem MTA^®^. Calcium carbonate and zirconium oxide were present in Medcem MTA^®^ and Biodentine™ while in ProRoot^®^ white MTA, bismuth oxide, calcium aluminium oxide and calcium sulphate were detected.

Calcium ions released (in μmol) by TSC in different pH media are summarized in Table 1.

The amount of calcium ions released by the TSC and pH rise (ΔpH) during consecutive elutions in distilled water are summarized in Table 2 and Table 3 respectively.

At all the time intervals, ΔpH and calcium ion release of each TSC differed not significantly between groups with the same exposed surface area (ESA) (*p* > 0.05). However, ΔpH and calcium ion release increased significantly with increasing ESA (*p* < 0.05). The above results indicated that ΔpH and calcium ion release were independent of the volume (Vol).

**Figure 2:**
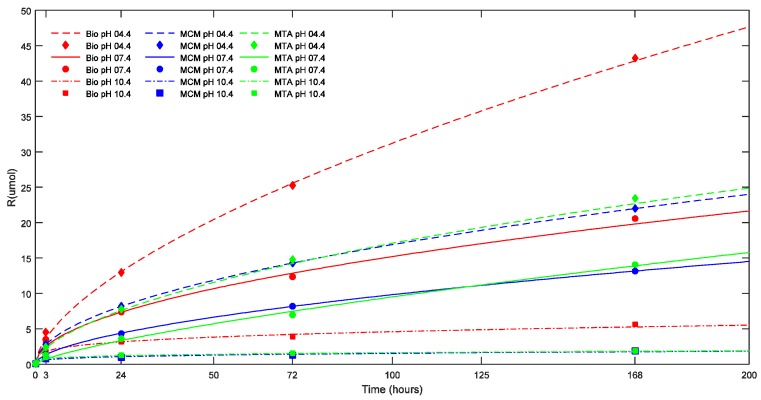
Effect of pH on the cumulative calcium ion release (R) of the TSCs studied (in μmoles) as a function of time. The points represent the measured values and the lines show the calculated values according to Equation (1). Bio: Biodentine™; MCM: Medcem MTA^®^; MTA: ProRoot^®^ white MTA.

**Figure 3:**
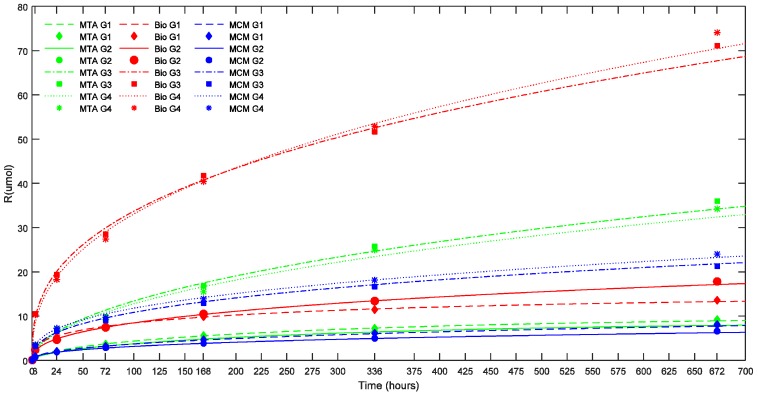
Effect of sample dimensions (Vol and ESA) on cumulative calcium ion release (R) of the TSCs studied (in μmoles) as a function of time. The points represent the measured values and the lines show the calculated values according to Equation (1). Bio: Biodentine™; MCM: Medcem MTA^®^; MTA: ProRoot^®^ white MTA. G1: Group 1 (1.5 × 5 mm; Vol: 8.835 mm^3^; ESA: 1.77 mm^2^); G2: Group 2 (1.5 × 10 mm; Vol: 17.67 mm^3^; ESA: 1.77 mm^2^); G3: Group 3 (3.5 × 5 mm; Vol: 48.11 mm^3^; ESA: 17.62 mm^2^); G4: Group 4 (3.5 × 10 mm; Vol: 96.22 mm^3^; ESA: 17.62 mm^2^).

At all the time intervals, there was no significant difference in ΔpH and calcium ion release between the TSC’s studied in groups with smaller ESA. In groups with larger ESA (17.62 mm^2^), Biodentine™ showed a significantly higher calcium ion release than ProRoot^®^ MTA and Medcem MTA^®^ (*p* < 0.05). On the other hand, ProRoo^®^t MTA demonstrated a significantly higher ΔpH and calcium ion release (*p* < 0.05) than Medcem MTA^®^ in groups with larger ESA after seven days. The effect of environmental pH, ESA and Vol on calcium ion release as evidenced by the statistical results can be illustrated in Figure 2 and Figure 3 where the cumulative calcium release (R in μmoles) is plotted versus the corresponding time in hours.

A non-linear regression analysis of the cumulative calcium ion release (μmol) as a function of time (t) (h) demonstrated that the data are best represented by Equation (1) on the basis of F statistic, corresponding *p* value, residual sum of squares, correlation coefficient (r^2^) and standard error of estimate.

R = R_∞_ (1 − e^−βt^)^δ^ (1)

The three parameters characteristic for Equation (1), R_∞_, β and δ are summarized in Table 4 and Table 5. R_∞_ denotes the maximum amount of calcium ions released at infinity while β is related to the rate at which R reaches the maximum. Equation (1) with δ = 1 represents a pure dissolution controlled process. For δ < 1 a correction is made for a deviation from a pure dissolution controlled release indicating a possible combination of a dissolution and diffusion controlled process taking place. Half-life time (t_1/2_) is calculated using Equation (1) as the time needed to release half of R_∞_ and is an indicator of the longevity of the reaction (in Table 4 and Table 5). The r^2^ value gives the goodness of fit of the equation (in Table 4 and Table 5).

**Figure 4:**
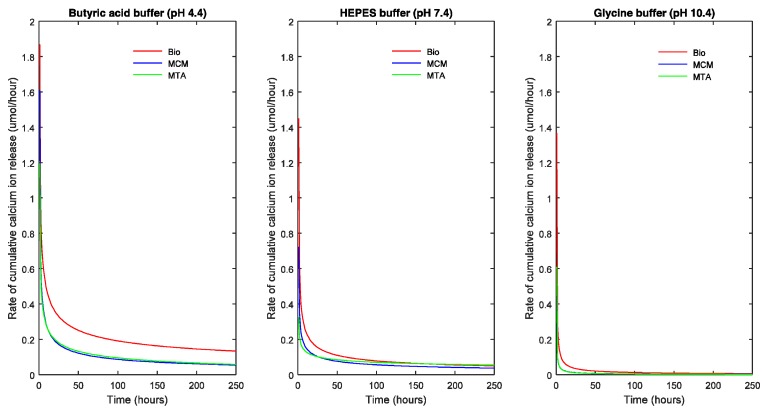
Cumulative calcium ion release rate of the TSCs as a function of the pH of the solution.

**Figure 5:**
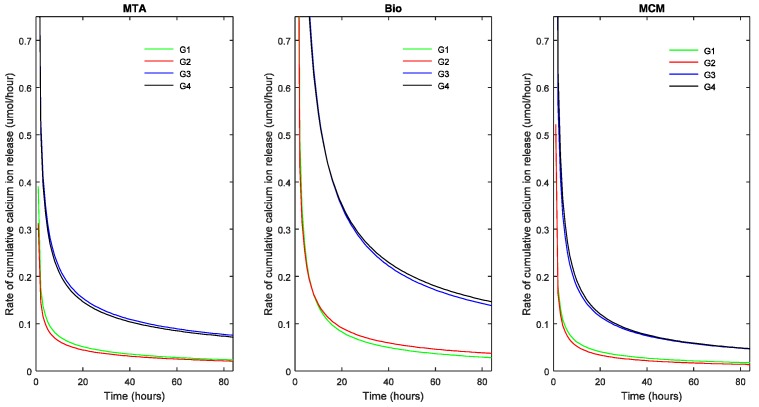
Cumulative calcium ion release rate of the TSCs studied as a function of sample dimensions. Bio: Biodentine™; MCM: Medcem MTA^®^; MTA: ProRoot^®^ white MTA. G1: Group 1 (1.5 × 5 mm; Vol: 8.835 mm^3^; ESA: 1.77 mm^2^); G2: Group 2 (1.5 × 10 mm; Vol: 17.67 mm^3^; ESA: 1.77 mm^2^); G3: Group 3 (3.5 × 5 mm; Vol: 48.11 mm^3^; ESA: 17.62 mm^2^); G4: Group 4 (3.5 × 10 mm; Vol: 96.22 mm^3^; ESA: 17.62 mm^2^).

In Figure 2 and Figure 3, the points represent the measured values and the full lines show the calculated values according to Equation (1). The release rate of each TSC in each pH milieu was calculated as the derivative (dCa/dt) and is illustrated as a function of pH and sample dimensions in Figure 4 and Figure 5, respectively.

The slope of the curve indicates the decrease in rate of R with respect to time. A steep fall in the curve would suggest a high decrease in reaction rate and flattening of the curve suggests a decreasing long-term release rate of calcium ions.

Calcium release profiles of the three TSCs showed certain general trends. Based on the parameters of Equation (1), the maximal R represented by R_∞_ was significantly higher in acidic solution followed by neutral and alkaline solution (*p* < 0.05) except for ProRoot^®^ white MTA which showed the highest R_∞_ in neutral solution (*p* < 0.05). In acidic and alkaline solution, Biodentine™ exhibited the highest R_∞_ (*p* < 0.05) while in a neutral solution, ProRoot^®^ white MTA exhibited the highest R_∞_ (*p* < 0.05).

In addition, for each TSC, the R_∞_ significantly differed between the four groups with different volumes (*p* < 0.05) indicating that R_∞_ was dependent on the volume of the biomaterial. This also holds true for the half-life (t_1/2_) (*p* < 0.05). Biodentine™ showed a significantly higher R_∞_ and t_1/2_ than ProRoot^®^ MTA (*p* < 0.05) and Medcem MTA^®^ (*p* < 0.05). The t_1/2_ value indicating the longevity of the calcium ion release was different for the TSCs studied and varied with respect to pH. Biodentine™ exhibited the highest t_1/2_ at acidic and neutral pH (*p* < 0.05) whereas Medcem MTA^®^ showed significantly higher t_1/2_ at alkaline pH (*p* < 0.05). The rate at which R reaches saturation was significantly different between groups with different ESA (*p* < 0.05). On the contrary, the volume did not play a significant role in the β parameter of Equation (1).

## 3. Discussion

The presence of calcium compounds in a dental material does not imply their dissociation and release by the materials after setting, because the setting reaction and the presence of other constituents can inhibit the release of calcium ions [28]. Another factor which could inhibit/promote calcium ion release from the materials is a change of the pH of the environment during and after the setting of TSCs. An acidic pH in the root canal could be due to the ingress of tissue fluids from dentinal tubules, exudate/pus from periapical area and from the acidification process caused by inflammatory cells. On the other hand, endodontic medicaments (calcium hydroxide with pH 12.4) and irrigants (such as 5% sodium hypochlorite with pH 11) with high pH can lead to a sustained high alkalinity in the root canal between appointments [29].

An acidic or alkaline pH has been shown to alter the physical and chemical properties of TSCs [30]. In an acidic environment, inability to set [31], impaired sealing ability [32], reduced surface hardness [31], decreased push-out bond strength [33] and diminished diametrical tensile strength [34] have been reported for MTA. Similarly, in an alkaline environment, decreased push-out bond strength [35], low surface hardness and increased porosity of white MTA has been reported in the literature [36]. In certain clinical applications, TSCs could be placed in an environment where inflammation may be present and consequently, the surface of the material will be exposed to low pH environment.

Different pH buffers and different types of acids have diverse effects on the calcium ion release due to a varying chelation effect. In the present study, we used butyric acid buffer, as butyric acid is a by-product of anaerobic bacterial metabolism and is clinically more relevant to simulate the conditions of periradicular infections [31]. In addition, in situations where the initiating and perpetuating factors of an inflammatory process are removed by appropriate treatment, the pH of the environment returns to normal within 168 h [31], justifying the duration of the present study.

The biological effects of TSCs are mostly attributed to the formation of calcium hydroxide during and after the setting of these hydraulic cements. Calcium hydroxide has proven properties of stimulating mineralization, protecting the pulp against thermoelectric stimuli and favoring antibacterial action [37]. However, to exert their biological and microbiological action, the calcium and hydroxide ions formed must be released. Calcium ions in particular, play a fundamental role in mineralization due to the induction of cellular migration and differentiation [38]. An increase in extracellular calcium ion concentration by 0.2–0.7 mM in cell culture media could elevate bone related gene expression in human dental pulp cells [39]. In the present study, calcium ion release resulted in a concentration of more than 0.2 mM for all cements in acidic solution and less than 0.2 mM for all cements in alkaline solution. However, in neutral solution, calcium ion release resulted in a concentration more than 0.2 mM at all time intervals only in the Biodentine™ group.

The increase of the calcium ion release observed in a solution of pH 4.4 could be due to increased solubility of the cement [40] in acidic medium. This leads to increased porosity of the cements and consequently diminished physical characteristics of the restorative material. Translation of these in vitro results clinically would imply the necessity to decrease inflammation and if possible, perform a two session treatment with placement of an alkaline medicament such as calcium hydroxide between the appointments [40]. Furthermore, these results also signify that a highly alkaline medium is harmful to calcium ion release and thorough irrigation of the root canal space between the usage of alkaline calcium hydroxide and the endodontic filling material is an absolute necessity to neutralize the pH of the root canal. Obturation of root canal space with TSCs is best preceded with thorough saline irrigation to neutralize the effect of other root canal irrigants.

At all measured time intervals, ΔpH and calcium ion release was dependent on the ESA while there was no correlation between the volume and the ΔpH or calcium ion release (Figure 3). ESA also determines the rate at which R reaches its maximum irrespective of the TSC variant. Compared to the present findings, a consistently higher pH and calcium ion release has been observed in previous studies using ProRoot^®^ MTA [4,23,26] and Biodentine™ [41]. This could be due to the significantly greater surface area of the specimens used in these studies: ProRoot^®^ MTA and Biodentine™ had an exposed surface area of 50.24 mm^2^ and 140.74 mm^2^ respectively. Similarly, studies that used a smaller ESA (0.79 mm^2^) exhibited lower calcium ion release in comparison to the present findings [42,43]. Moreover, these studies used distilled/deionized water at a pH ranging from 6.8–7.0 compared to 5.7 used in the present study. Moreover, the samples were not always immersed in water after the setting time.

The R_∞_ and the t_1/2_ was significantly different in groups with different volumes. These were the only two factors dependent on the volume of the TSC. The presence of adequate calcium hydroxide for dissociation on the exposed surface of the TSC sample might be an explanation. Apparently, the calcium is leaching from a thin surface layer of the TSC. The effect of the ESA on the Ca release however increased with increasing volume for ProRoot^®^ MTA and Medcem MTA^®^. On the long-term, diffusion of calcium from deep within the sample might occur for all TSCs, as indicated by the R_∞_ and t_1/2_.

In specimens with larger surface area (17.62 mm^2^), R of Biodentine™ was higher than ProRoot^®^ MTA and Medcem MTA^®^ (Table 5). Increased R of Biodentine™ could be responsible for the induction of more rapid calcified tissue deposition compared to MTA [44]. Reasons for increased R with Biodentine™ could be the presence of pure synthetic tricalcium silicate as identified with XRD analysis [45], absence of dicalcium silicate and tricalcium aluminate, and addition of calcium chloride to the liquid component. The use of calcium chloride in the liquid component resulting in faster setting of Biodentine™ not only increases the calcium ion release by increased calcium hydroxide formation but also contributes to the calcium ion release by unreacted calcium chloride. The absence of dicalcium silicate and tricalcium aluminate in Biodentine™ has been confirmed by powder characterization using XRD. This could explain the decreased calcium ion release in ProRoot^®^ MTA and Medcem MTA^®^, because dicalcium silicate and tricalcium aluminate respectively form minimal and no calcium hydroxide upon cement hydration. The compositional differences in the three TSCs analyzed could be a major factor responsible for the difference in calcium ion release. The other factors that are expected to contribute to the increased R of Biodentine™ are smaller particle size [46] and increased solubility [47].

Only positive ΔpH values were obtained at all the time intervals irrespective of the TSCs being evaluated, showing that the eluted components were of alkaline nature. This is due to the formation of calcium hydroxide during the setting and maturation of the tricalcium silicate based cements. A correlation between ΔpH and Log[Ca] was generally observed for ProRoot^®^ MTA and Medcem MTA^®^. However, such correlation was sporadic in the case of Biodentine™. These findings could also be explained by the presence of calcium chloride in the liquid component of Biodentine™.

In vitro studies and animal models evaluating TSCs for calcium release and mineralization should consider the exposed surface area and volume of TSC to be used when devising the methodology. The present results could be partially translated to a clinical environment, where TSCs would release calcium and hydroxide ions when in contact with an aqueous environment. The exposed surface in the present study was either 1.77 mm^2^ or 17.62 mm^2^. A clinical analogy would be that for a mature permanent maxillary central incisor requiring pulpotomy, the average surface area of the pulp tissue exposed to the TSC would be approximately 1.86 mm^2^. Clinical translation of the current results would mean that in pulp capping or pulpotomy procedure, the amount of calcium ions released would not be dependent on the TSC variant as the ESA would be minimal in these cases. On the other hand, treatment modalities such as root-end filling or treatment of immature teeth after trauma/severe infection, which possess a large surface area of lesion would benefit from increased calcium ion release when using Biodentine™ compared to ProRoot^®^ MTA or Medcem MTA^®^. Mineralization and dentine bridge formation in pulp capping and pulpotomy procedures could be promoted by increasing the ESA within the clinical limitations. In clinical procedures such as obturation of root canal and perforation repair, an increased volume of the TSC would help in sustained long-term release of calcium ions.

The evaluation of such properties of the recently developed materials is fundamental [42], because the effect of compositional differences of each TSC variant on its calcium ion release properties is still not precisely known. In addition, the relationship between extracellular calcium ion concentration and cell behavior need to be studied extensively. These future studies could help modify the composition of the currently available TSCs to achieve optimum calcium release for faster mineralization while maintaining minimal calcium related cytotoxicity. Current direction of research employs various techniques to alter the calcium content and release of commercial biomaterials. The inclusion of other calcium compounds such as calcium phosphate phase [48], calcium oxide [20], calcium sulphate [49], calcium carbonate [42], calcium chloride [50] and calcium hydroxide [22] are among the few published techniques. The use of pre/mixed TSCs [51] and light cured TSCs [24] are other options that would modify calcium release.

## 4. Materials and Methods

ProRoot^®^ white MTA (Dentsply, Tulsa dental specialties, Tulsa, OK, USA) and Medcem MTA^®^ GmbH (Weinfelden, Switzerland) were hand mixed on a sterile glass slab with a water to powder ratio of 0.35 with sterile water. Biodentine™ (Septodont, Saint Maur des Fosses, France) was mixed in a capsule mixing device (3M™ ESPE™ RotoMix™, Seefeld, Germany) based on rotation mixing principle with the liquid supplied by the manufacturer. The water to powder ratio was 0.35 and the liquid component of Biodentine™ is known to contain calcium chloride and hydrosoluble polymer.

### 4.1. XRD Procedure and Analysis

XRD patterns of the powder component of each TSC was collected between 2 degrees to 60 degrees 2θ and scanned at steps of 0.02 degrees lasting 4 s each using an X-ray diffractometer (PW1830 Philips diffractometer, PANalytical, Eindhoven, Netherlands) with CuKα radiation (λ = 0.154 nm). To identify crystalline compounds, the XRD patterns were matched using the Powder Diffraction File (PDF) database of the International Centre for Diffraction Data (ICDD).

### 4.2. Sample Preparation for Effect of ESA and Vol on Calcium Ion Release and pH

The cement samples were randomly allotted to one of four groups (*n* = 6) with different dimensions and condensed into cylindrical acrylic resin (Plexiglas) vessels. Obturation efficiency was verified by radiographs and weight. The four groups had varying length (L), diameter (ϕ), ESA and Vol are shown in Table 6.

The groups were designed in such a way that groups 1 and 2 had a smaller ESA compared to groups 3 and 4. The Vol increased from groups 1 to 4. Immediately after the initial setting time, each sample was separately immersed in 10 mL deionized ultra-pure water (MilliQ Academic, Millipore, Bedford, MA, USA) and gently shaken at 37 °C. After 3 h, 1, 3, 7, 14 and 28 days the samples were taken out of the solution, blotted dry with filter paper and transferred to 10 mL of fresh deionized water for further elution. pH and calcium content of the solutions were measured.

### 4.3. Sample Preparation for Effect of pH on Calcium Ion Release

The cements were condensed gently in cylindrical Plexiglas vessels of internal diameter 1.5 mm and length 10 mm corresponding to an exposed surface area of 1.77 mm^2^ and volume of 17.67 mm^3^ (*n* = 18 per cement group). Three buffer solutions were prepared: 20 mM glycine (Merck Millipore, Darmstadt, Germany) buffered to a pH of 10.4, 20 mM HEPES (Fluka, Sigma Aldrich, Diegem, Belgium) buffered to a pH of 7.4 and 20 mM butyric acid (Merck Millipore, Darmstadt, Germany) buffered to a pH of 4.4. Cylindrical Plexiglass vessels were randomly allocated to each group of the pH buffer solutions (*n* = 6 per cement group). Each Plexiglass vessel was placed in 10 mL of respective pH medium in a PE flask immediately after their setting time. The sealed flasks were stored at 37 °C on a shaker. At 3 h, 1, 3 and 7 days, the Plexiglass vessels were moved to new flasks with fresh buffer solution and the liquid in which they were previously kept was subjected to calcium analysis.

### 4.4. pH and Calcium Ion Release Measurement

The pH of the solutions were measured with a pH glass electrode (Primatrode, Metrohm, Switzerland). The concentration of calcium ions was measured using an atomic absorption spectrophotometer (Varian SpectrAA-30, Agilent Technologies, Santa Clara, CA, USA) equipped with a specific calcium hollow cathode lamp (Agilent Technologies, Santa Clara, CA, USA). The spectrophotometer was operated following the manufacturer’s instructions using a wavelength of 422.70 nm, slit width of 0.2 nm and a current of 10 mA for the lamp.

An air/acetylene flame and 0.2 M HCl/4000 mg/L La (LaCl_3_) as buffer were used. Buffer was added to both standard and diluted sample solutions to eliminate interference and to match the pH of all solutions. The results were calculated using a calibration curve established on the basis of standard solutions (1–5 ppm Ca). Standards were prepared from a 1000 mg/L calcium in HCl solution (as CaCl_2_, Titrisol, Merck Millipore, Darmstadt, Germany). The relative uncertainties at the 95% confidence level on the amount of calcium was determined as 2%.

### 4.5. Calcium Ion Release Analysis

The cumulative release of calcium ions was calculated. Regression analysis, curve fitting and graphs were performed using Matlab 8.0 (The Mathworks Inc., Natick, MA, USA) software. Data were subjected to statistical analysis (analysis of variance) and individual comparisons were performed by using the Bonferroni method at a significance level of *p* < 0.05 using Statistical Package for Social Sciences (SPSS) v21.0 (IBM Corp., Armonk, NY, USA).

## 5. Conclusions

In the short-term, pH and calcium ion release depend on the ESA, whereas the maximum amount of calcium ions released at infinity (R_∞_) is dependent on the volume of TSC. For small ESA (1.77 mm^2^), there was no difference in the calcium ion release between the three TSCs. In the higher ESA group (17.62 mm^2^), calcium ion release was in the order of Biodentine™ > ProRoot**^®^** white MTA > Medcem MTA^®^. All the three TSCs released more calcium ions in acidic solution when compared to neutral solution which in turn resulted in higher calcium release than alkaline solution. Biodentine™ released more calcium ions than ProRoot^®^ white MTA and Medcem MTA^®^ irrespective of the pH of the environment. An alkaline pH in the root canal would be detrimental to the calcium ion release of the tricalcium silicate based endodontic cements.

## Figures and Tables

**Table 1:** Amount of calcium ions released (in μmol) with corresponding standard deviation (*n* = 6) between parentheses of the TSC variants studied as a function of pH at different time intervals.

Group	TSC Variant (1.5 × 10 mm)	3 h	24 h	72 h	168 h
pH 4.4	ProRoot^®^ white MTA	2.41 (0.68)	5.39 (0.71)	6.96 (0.82)	8.69 (0.70)
	Medcem MTA^®^	2.83 (1.27)	5.39 (0.19)	6.04 (0.19)	7.79 (0.40)
	Biodentine™	4.49 (1.21)	8.49 (0.66)	12.31 (1.01)	17.98 (1.44)
pH 7.4	ProRoot^®^ white MTA	1.23 (0.09)	2.40 (0.22)	3.31 (0.09)	7.15 (0.50)
	Medcem MTA^®^	1.38 (0.23)	2.97 (0.30)	3.81 (0.24)	4.97 (0.17)
	Biodentine™	3.54 (0.60)	3.80 (0.34)	5.02 (0.72)	8.19 (0.71)
pH 10.4	ProRoot^®^ white MTA	0.92 (0.19)	0.39 (0.10)	0.28 (0.05)	0.30 (0.05)
	Medcem MTA^®^	0.75 (0.12)	0.21 (0.03)	0.34 (0.03)	0.55 (0.09)
	Biodentine™	2.13 (0.91)	1.07 (0.31)	0.72 (0.65)	1.74 (0.82)

*Legend:* pH 4.4, 7.4 and 10.4 indicate the pH of butyric acid buffer, HEPES buffer and glycine buffer respectively.

**Table 2:** Amount of calcium ions released (in μmol) by the TSCs as a function of sample dimensions (varying Vol and ESA) with corresponding standard deviation (*n* = 6) between parentheses at different time intervals.

Group	Vol (mm^3^)	ESA (mm^2^)	TSC Variant	3 h	24 h	72 h	168 h	336 h	672 h
**1.5 × 5 mm (G1)**	8.835	1.77	ProRoot^®^ white MTA	0.75 (0.03)	1.34 (0.45)	1.55 (0.06)	1.89 (0.12)	1.69 (0.10)	1.91 (0.13)
			Medcem MTA^®^	0.81 (0.07)	1.20 (0.04)	1.17 (0.03)	1.36 (0.04)	1.50 (0.13)	2.06 (0.10)
			Biodentine™	2.91 (0.86)	2.38 (0.27)	2.62 (0.32)	1.97 (0.33)	1.55 (0.12)	2.12 (0.12)
**r1.5 × 10 mm (G2)**	17.67	1.77	ProRoot^®^ white MTA	0.67 (0.03)	1.13 (0.09)	1.31 (0.11)	1.69 (0.08)	1.69 (0.15)	1.63 (0.25)
			Medcem MTA^®^	0.76 (0.07)	1.04 (0.12)	0.97 (0.11)	1.07 (0.10)	1.07 (0.14)	1.63 (0.12)
			Biodentine™	2.33 (0.09)	2.32 (0.26)	2.69 (0.12)	3.10 (0.09)	3.04 (0.17)	4.40 (1.27)
**3.5 × 5 mm (G3)**	48.11	17.62	ProRoot^®^ white MTA	3.52 (0.75)	3.52 (0.64)	2.85 (0.76)	6.93 (1.07)	8.92 (0.33)	10.23 (0.52)
			Medcem MTA^®^	3.25 (0.37)	3.29 (0.50)	2.45 (0.45)	3.89 (1.26)	3.76 (0.99)	4.63 (2.02)
			Biodentine™	10.50 (1.40)	8.93 (1.19)	9.17 (1.16)	13.06 (1.42)	9.92 (1.94)	19.52 (1.03)
**3.5 × 10 mm (G4)**	96.22	17.62	ProRoot^®^ white MTA	3.19 (0.44)	4.04 (0.45)	1.89 (0.13)	6.42 (0.45)	9.42 (0.38)	9.17 (0.86)
			Medcem MTA^®^	3.42 (0.36)	3.76 (0.71)	2.54 (0.37)	4.03 (0.72)	4.20 (1.19)	5.94 (2.55)
			Biodentine™	10.32 (1.02)	7.96 (0.68)	9.18 (0.86)	12.98 (0.89)	12.44 (2.12)	21.21 (0.94)

**Table 3:** ΔpH as a function of sample dimensions (varying Vol and ESA) with corresponding standard deviation (*n* = 6) between parentheses at different time intervals.

Group	Vol (mm^3^)	ESA (mm^2^)	TSC Variant	3 h	24 h	72 h	168 h	336 h	672 h
**1.5 × 5 mm (G1)**	8.835	1.77	ProRoot^®^ white MTA	0.64 (0.01)	0.76 (0.04)	1.43 (0.02)	1.51 (0.17)	0.56 (0.08)	0.86 (0.10)
			Medcem MTA^®^	0.27 (0.01)	0.23 (0.12)	1.35 (0.05)	1.31 (0.04)	1.46 (0.05)	1.05 (0.05)
			Biodentine™	1.00 (0.36)	1.05 (0.06)	1.07 (0.18)	1.38 (0.20)	0.37 (0.01)	0.63 (0.05)
**1.5 × 10 mm (G2)**	17.67	1.77	ProRoot^®^ white MTA	0.96 (0.25)	1.01 (0.17)	1.42 (0.35)	1.32 (0.43)	1.35 (0.12)	1.50 (0.06)
			Medcem MTA^®^	0.12 (1.24)	0.93 (0.94)	1.35 (0.30)	1.53 (0.57)	1.57 (0.64)	1.32 (1.06)
			Biodentine™	1.15 (0.35)	0.76 (0.70)	0.65 (0.24)	1.83 (0.12)	1.45 (0.32)	1.60 (0.05)
**3.5 × 5 mm (G3)**	48.11	17.62	ProRoot^®^ white MTA	3.56 (0.65)	4.11 (0.32)	2.53 (0.34)	3.91 (0.64)	4.93 (0.10)	5.18 (0.08)
			Medcem MTA^®^	2.65 (1.12)	2.99 (0.80)	1.86 (0.81)	3.14 (0.53)	3.95 (0.58)	3.67 (0.94)
			Biodentine™	2.07 (0.15)	3.07 (0.50)	3.95 (0.66)	4.71 (0.12)	4.62 (0.61)	5.38 (0.05)
**3.5 × 10 mm (G4)**	96.22	17.62	ProRoot^®^ white MTA	4.09 (0.25)	4.27 (0.17)	3.32 (0.35)	4.24 (0.43)	4.89 (0.12)	5.13 (0.05)
			Medcem MTA^®^	2.99 (1.24)	3.22 (0.94)	2.59 (0.30)	3.35 (0.57)	3.82 (0.64)	4.01 (1.06)
			Biodentine™	1.10 (0.35)	2.91 (0.70)	4.18 (0.24)	4.67 (0.12)	4.87 (0.32)	5.32 (0.05)

**Table 4:** Values of the parameters of Equation (1), t_1/2_ and r^2^ with corresponding standard deviation (*n* = 6) between parentheses for the cumulative calcium release of the TSCs as a function of pH.

Group	TSC Variant	R_∞_	β × 10^4^	δ	t_1/2_ (days)	r^2^
**pH 4.4**	ProRoot^®^ white MTA	68.67 (0.58)	9.65 (0.84)	0.58 (0.00)	15.78 (1.20)	0.98 (0.04)
	Medcem MTA^®^	197.33 (0.51)	0.82 (0.01)	0.51 (0.00)	151.92 (1.77)	0.98 (0.03)
	Biodentine™	993.66 (0.61)	0.40 (0.01)	0.61 (0.00)	463.19 (11.05)	0.99 (0.01)
**pH 7.4**	ProRoot^®^ white MTA	792.74 (0.73)	0.24 (0.01)	0.73 (0.00)	856.16 (23.31)	0.98 (0.00)
	Medcem MTA^®^	139.40 (0.57)	0.93 (0.11)	0.57 (0.00)	156.79 (13.77)	0.99 (0.00)
	Biodentine™	520.67 (0.51)	0.10 (0.01)	0.51 (0.00)	1259.90 (31.09)	0.98 (0.02)
**pH 10.4**	ProRoot^®^ white MTA	8.45 (0.21)	0.05 (0.00)	0.21 (0.00)	363.05 (7.10)	0.90 (0.11)
	Medcem MTA^®^	14.39 (0.26)	0.01 (0.00)	0.26 (0.00)	1668.10 (75.16)	0.93 (0.04)
	Biodentine™	37.67 (0.26)	0.03 (0.00)	0.26 (0.00)	917.92 (42.99)	0.82 (0.09)

***Legend*:** Values and corresponding standard deviation in column 3, 4 and 5 are the parameters of the cumulative calcium release according to Equation (1): R = R_∞_ (1 − e^−βt^)^δ^. t_1/2_ represents the half-life time (days) of the calcium release and r^2^ (correlation coefficient) values represent the goodness of fit of Equation (1).

**Table 5:** Values of the parameters of the Equation (1), t_1/2_ and r^2^ with corresponding standard deviation (*n* = 6) between parentheses for the cumulative calcium release of the TSCs as a function of sample dimensions.

Group	Vol (mm^3^)	ESA (mm^2^)	TSC Variant	R_∞_	β × 10^4^	δ	t_1/2_ (days)	r^2^
**1.5 × 5 mm (G1)**	8.835	1.77	ProRoot^®^ white MTA	10.11 (0.25)	23.42 (0.63)	0.54 (0.01)	5.73 (0.18)	0.99 (0.00)
			Medcem MTA^®^	11.49 (0.42)	7.72 (0.66)	0.44 (0.01)	12.37 (0.99)	0.99 (0.00)
			Biodentine™	14.67 (0.41)	19.53 (6.62)	0.32 (0.00)	2.89 (1.09)	0.99 (0.01)
**1.5 × 10 mm (G2)**	17.67	1.77	ProRoot^®^ white MTA	9.04 (0.35)	22.97 (0.93)	0.55 (0.01)	6.12 (0.28)	0.99 (0.00)
			Medcem MTA^®^	9.61 (0.37)	6.09 (1.01)	0.39 (0.01)	13.24 (2.23)	0.99 (0.00)
			Biodentine™	26.83 (0.52)	5.93 (0.61)	0.40 (0.01)	14.00 (1.28)	0.99 (0.01)
**3.5 × 5 mm (G3)**	48.11	17.62	ProRoot^®^ white MTA	70.43 (0.47)	4.32 (0.81)	0.52 (0.01)	30.78 (6.02)	0.99 (0.01)
			Medcem MTA^®^	37.33 (0.52)	4.34 (0.81)	0.39 (0.02)	18.47 (3.47)	0.93 (0.07)
			Biodentine™	526.17 (0.52)	0.06 (0.01)	0.37 (0.00)	1242.90 (118.64)	0.99 (0.01)
**3.5 × 10 mm (G4)**	96.22	17.62	ProRoot^®^ white MTA	64.333 (0.52)	4.67 (0.20)	0.52 (0.01)	27.63 (1.32)	0.98 (0.00)
			Medcem MTA^®^	126.50 (0.55)	0.13 (0.01)	0.36 (0.01)	501.00 (61.99)	0.92 (0.10)
			Biodentine™	711.49 (0.54)	0.04 (0.00)	0.40 (0.00)	1820.70 (152.02)	0.99 (0.00)

***Legend*:** Values and corresponding standard deviation in column 3, 4 and 5 are the parameters of the cumulative calcium release according to Equation (1): R = R_∞_ (1 − e^−βt^)^δ^. t_1/2_ represents the half-life time (days) of the calcium release and r^2^ (correlation coefficient) values represent the goodness of fit of Equation (1).

**Table 6:** Outline of the four groups with different ESA and Vol.

Groups	Length (L)	Diameter (ϕ)	ESA	Vol
Group 1 (G1)	5 mm	1.5 mm	1.77 mm^2^	8.835 mm^3^
Group 2 (G2)	10 mm	1.5 mm	1.77 mm^2^	17.67 mm^3^
Group 3 (G3)	5 mm	3.5 mm	17.62 mm^2^	48.11 mm^3^
Group 4 (G4)	10 mm	3.5 mm	17.62 mm^2^	96.22 mm^3^
