# Correction: Rajasekharan, S., et al. Effect of Exposed Surface Area, Volume and Environmental pH on the Calcium Ion Release of Three Commercially Available Tricalcium Silicate Based Dental Cements. *Materials* 2018, *11*, 123

**DOI:** 10.3390/ma14020340

**Published:** 2021-01-12

**Authors:** Sivaprakash Rajasekharan, Chris Vercruysse, Luc Martens, Ronald Verbeeck

**Affiliations:** 1 Department of Pediatric Dentistry & Special Care, PAECOMEDIS Research Cluster, Ghent University, 9000 Ghent, Belgium; luc.martens@ugent.be; 2 Biomaterials Group, Department of Basic Medical Sciences, Ghent University, 9000 Ghent, Belgium; chris.vercruysse@ugent.be (C.V.); Ronald.Verbeeck@ugent.be (R.V.)

The authors wish to make the following correction to the paper [1]:

**Conflicts of Interest:** The authors would like to report non-support (i.e., cement materials free of charge) from Septodont, France and MedCem (Switserland) during the conduct of the study. Outside of the submitted work, the authors received materials free of charge for educational courses and Martens, Rajasekharan, and Cauwels report personal fees (honoraria/lecture fees) and research grants from Septodont, France negotiated with Ghent University. The funders had no role in the design of the studies; in the collection, analyses, or interpretation of data; in the writing of the manuscripts, or in the decision to publish the results.

The authors would like to apologize for any inconvenience caused to the readers by these changes.
